# The genome sequence of the wood mouse, *Apodemus sylvaticus *(Linnaeus, 1758)

**DOI:** 10.12688/wellcomeopenres.20001.1

**Published:** 2023-10-12

**Authors:** Sarah C. L. Knowles, Aura Raulo, University of Oxford and Wytham Woods Genome Acquisition Lab, Darwin Tree of Life Barcoding collective, Wellcome Sanger Institute Tree of Life programme, Wellcome Sanger Institute Scientific Operations: DNA Pipelines collective, Tree of Life Core Informatics collective, Darwin Tree of Life Consortium

**Affiliations:** 1 University of Oxford, Oxford, England, UK

**Keywords:** Apodemus sylvaticus, wood mouse, genome sequence, chromosomal, Rodentia

## Abstract

We present a genome assembly from an individual male *Apodemus sylvaticus* (the wood mouse; Chordata; Mammalia; Rodentia; Muridae). The genome sequence is 2,889.8 megabases in span. Most of the assembly is scaffolded into 25 chromosomal pseudomolecules, including the X and Y sex chromosomes. The mitochondrial genome has also been assembled and is 16.31 kilobases in length.

## Species taxonomy

Eukaryota; Metazoa; Eumetazoa; Bilateria; Deuterostomia; Chordata; Craniata; Vertebrata; Gnathostomata; Teleostomi; Euteleostomi; Sarcopterygii; Dipnotetrapodomorpha; Tetrapoda; Amniota; Mammalia; Theria; Eutheria; Boreoeutheria; Euarchontoglires; Glires; Rodentia; Myomorpha; Muroidea; Muridae; Murinae; Apodemus; Sylvaemus group (Linnaeus, 1758) (NCBI:txid10129).

## Background

**Figure 1.**
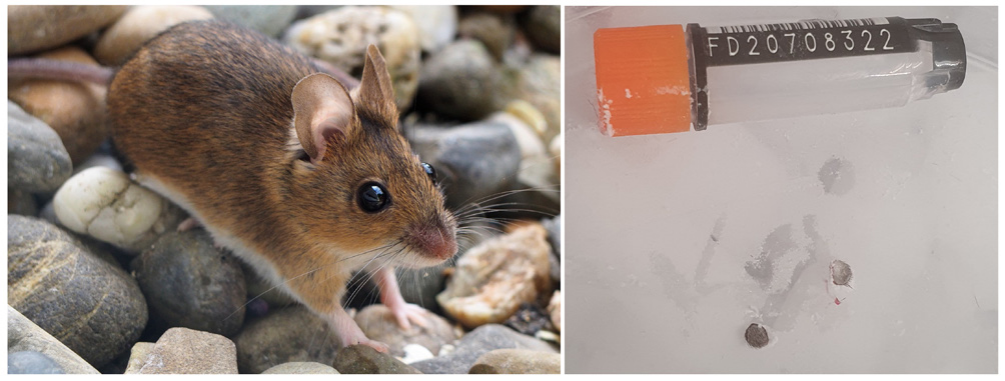
**A**. Photograph of a juvenile *Apodemus sylvaticus* (by BlueBreezeWiki) (not the specimen used for genome sequencing). **B**. Ear clippings of individual mApoSyl1 used for genome sequencing and Hi-C data.

The wood mouse (or long-tailed field mouse), *Apodemus sylvaticus* ( Figure 1), is an extremely common and widespread species of mouse with a broad geographic range spanning most of Europe as well as parts of northern Africa, from sea level to nearly 2 km in altitude ( IUCN, 2022). Wood mice inhabit a wide variety of habitats, including woodlands, grassland, hedgerows, urban environments and gardens, arable land, moorland and sand dunes. Throughout much of their range, wood mice are sympatric with the closely related species *Apodemus flavicollis* (yellow-necked mouse) and can be distinguished from this species by the lack of a continuous band of brown fur across the neck and typically smaller adult size. Wood mice are flexible omnivores, though are usually primarily granivorous, eating seeds from trees including oak, beech, ash, hawthorn and sycamore, but also invertebrates, fruit and fungi depending on availability ( Khammes & Aulagnier, 2007; Watts, 1968; Zubaid & Gorman, 1991).

Wood mice build extensive underground burrows in which they sleep and cache food ( Jennings, 1975). They are almost entirely nocturnal, emerging to make foraging trips throughout the night ( Wolton, 1985). They have small, overlapping home ranges that vary in size and overlap with sex and the stage of the breeding season ( Godsall *et al.*, 2014; Wolton, 1985) Their mating system is promiscuous, with males typically mating multiple females and litters often containing pups from multiple males ( Booth *et al.*, 2007).

Wood mice have become a key model species in ecology and evolutionary biology due to their abundance, ease of capture, and ability to be maintained in captivity. For example, they are frequently used in studies of parasite and microbiome ecology ( Knowles *et al.*, 2013; Marsh *et al.*, 2022; Raulo *et al.*, 2021; Sweeny *et al.*, 2021), eco-immunology ( Babayan *et al.*, 2018; Clerc *et al.*, 2019), sexual selection ( Moore *et al.*, 2002) and behavioural ecology ( Stopka & Macdonald, 2003). They are also of interest as relatively anthropophilic reservoirs of zoonotic pathogens ( Borg *et al.*, 2017; Crouch *et al.*, 1995). This species commonly undergoes dramatic seasonal bottlenecks, whereby over 80% of the population can die between breeding seasons ( Flowerdew, 1985). Despite these frequent bottlenecks, it has been shown that wood mouse populations can be relatively stable in genetic composition ( Booth *et al.*, 2005).

The genome of the wood mouse, *Apodemus sylvaticus*, was sequenced as part of the Darwin Tree of Life Project, a collaborative effort to sequence all named eukaryotic species in the Atlantic Archipelago of Britain and Ireland. Here we present a chromosomally complete genome sequence for *Apodemus sylvaticus*, based on ear clipping samples from one male specimen from Wytham Woods, Oxfordshire, UK.

## Genome sequence report

The genome was sequenced from one male *Apodemus sylvaticus* ( Figure 1) caught in Wytham Woods, Oxfordshire, UK (51.77, –1.34). A total of 25-fold coverage in Pacific Biosciences single-molecule HiFi long reads and 16-fold coverage in 10X Genomics read clouds were generated. Primary assembly contigs were scaffolded with chromosome conformation Hi-C data. Manual assembly curation corrected 138 missing joins or misjoins and removed 5 haplotypic duplications, reducing the scaffold number by 11.39%.

**Figure 2.**
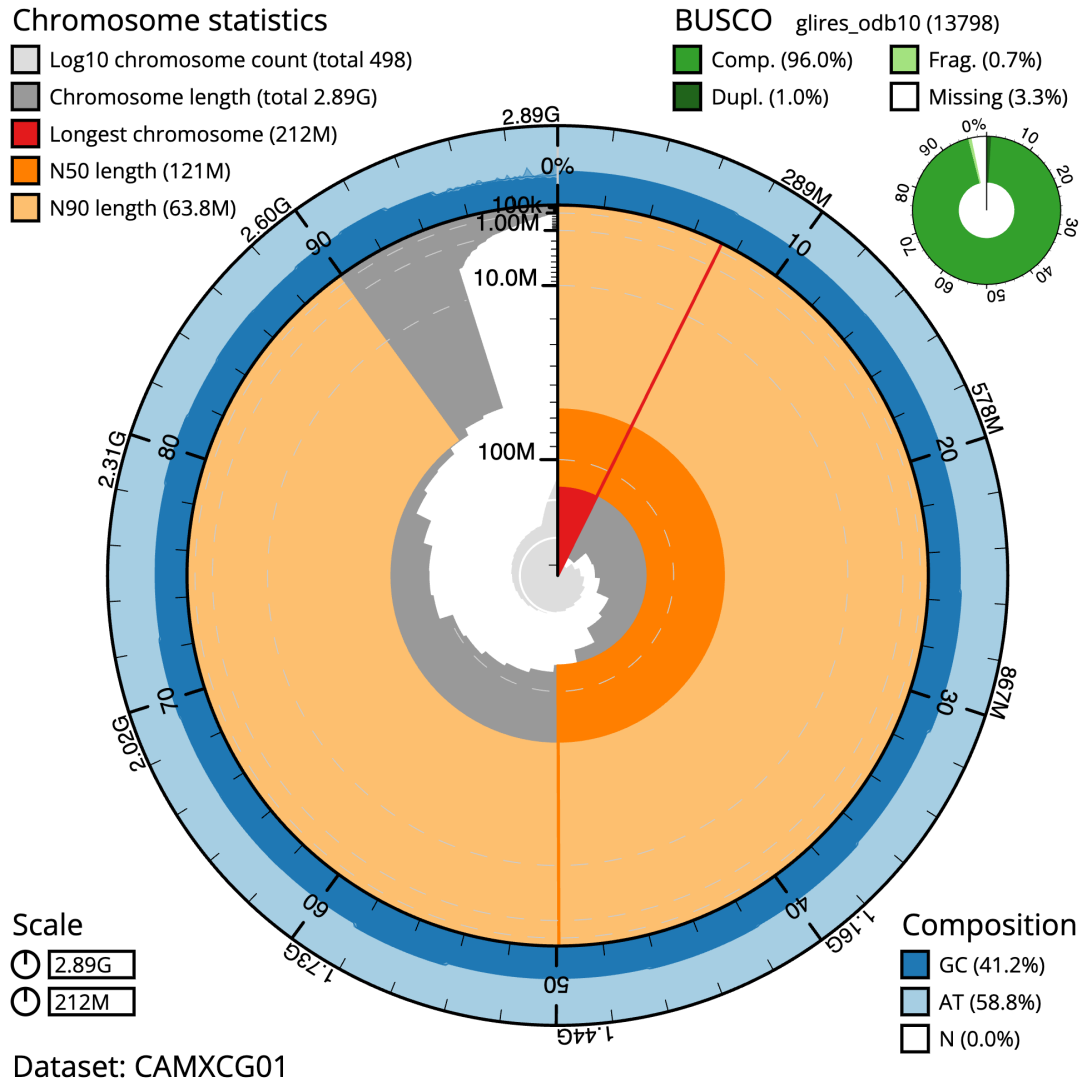
Genome assembly of *Apodemus sylvaticus*, mApoSyl1.1: metrics. The BlobToolKit Snailplot shows N50 metrics and BUSCO gene completeness. The main plot is divided into 1,000 size-ordered bins around the circumference with each bin representing 0.1% of the 2,889,801,511 bp assembly. The distribution of scaffold lengths is shown in dark grey with the plot radius scaled to the longest scaffold present in the assembly (211,764,336 bp, shown in red). Orange and pale-orange arcs show the N50 and N90 scaffold lengths (120,938,251 and 63,776,737 bp), respectively. The pale grey spiral shows the cumulative scaffold count on a log scale with white scale lines showing successive orders of magnitude. The blue and pale-blue area around the outside of the plot shows the distribution of GC, AT and N percentages in the same bins as the inner plot. A summary of complete, fragmented, duplicated and missing BUSCO genes in the glires_odb10 set is shown in the top right. An interactive version of this figure is available at https://blobtoolkit.genomehubs.org/view/mApoSyl1.1/dataset/CAMXCG01/snail.

The final assembly has a total length of 2889.8 Mb in 497 sequence scaffolds with a scaffold N50 of 120.9 Mb ( Table 1). Most (95.59%) of the assembly sequence was assigned to 25 chromosomal-level scaffolds, representing 23 autosomes and the X and Y sex chromosomes. Chromosome-scale scaffolds confirmed by the Hi-C data are named in order of size ( Figure 2– Figure 5; Table 2). While not fully phased, the assembly deposited is of one haplotype. Contigs corresponding to the second haplotype have also been deposited. The mitochondrial genome was also assembled and can be found as a contig within the multifasta file of the genome submission.

**Table 1.** Genome data for *Apodemus sylvaticus*, mApoSyl1.1.

Project accession data		
Assembly identifier	mApoSyl1.1	
Species	*Apodemus sylvaticus*	
Specimen	mApoSyl1	
NCBI taxonomy ID	10129	
BioProject	PRJEB53556	
BioSample ID	SAMEA7702024	
Isolate information	mApoSyl1, male: ear clip (DNA sequencing) mApoSyl2, female: ear clip (Hi-C scaffolding)	
Assembly metrics *		*Benchmark*
Consensus quality (QV)	55.9	*≥ 50*
*k*-mer completeness	99.99%	*≥ 95%*
BUSCO **	C:96.0%[S:95.0%,D:1.0%], F:0.7%,M:3.3%,n:13,798	*C ≥ 95%*
Percentage of assembly mapped to chromosomes	95.59%	*≥ 95%*
Sex chromosomes	X and Y chromosomes	*localised homologous pairs*
Organelles	Mitochondrial genome assembled	*complete single alleles*
Raw data accessions		
PacificBiosciences SEQUEL II	ERR9863246, ERR9854835, ERR9854836, ERR9871432	
10X Genomics Illumina	ERR9866441, ERR9866438, ERR9866439, ERR9866440	
Hi-C Illumina	ERR9866442	
Genome assembly		
Assembly accession	GCA_947179515.1	
*Accession of alternate haplotype*	GCA_947179525.1	
Span (Mb)	2,889.8	
Number of contigs	1,627	
Contig N50 length (Mb)	4.4	
Number of scaffolds	497	
Scaffold N50 length (Mb)	120.9	
Longest scaffold (Mb)	211.8	

* Assembly metric benchmarks are adapted from column VGP-2020 of “Table 1: Proposed standards and metrics for defining genome assembly quality” from ( Rhie *et al.*, 2021). ** BUSCO scores based on the glires_odb10 BUSCO set using v5.3.2. C = complete [S = single copy, D = duplicated], F = fragmented, M = missing, n = number of orthologues in comparison. A full set of BUSCO scores is available at https://blobtoolkit.genomehubs.org/view/mApoSyl1.1/dataset/CAMXCG01/busco.

**Figure 3.**
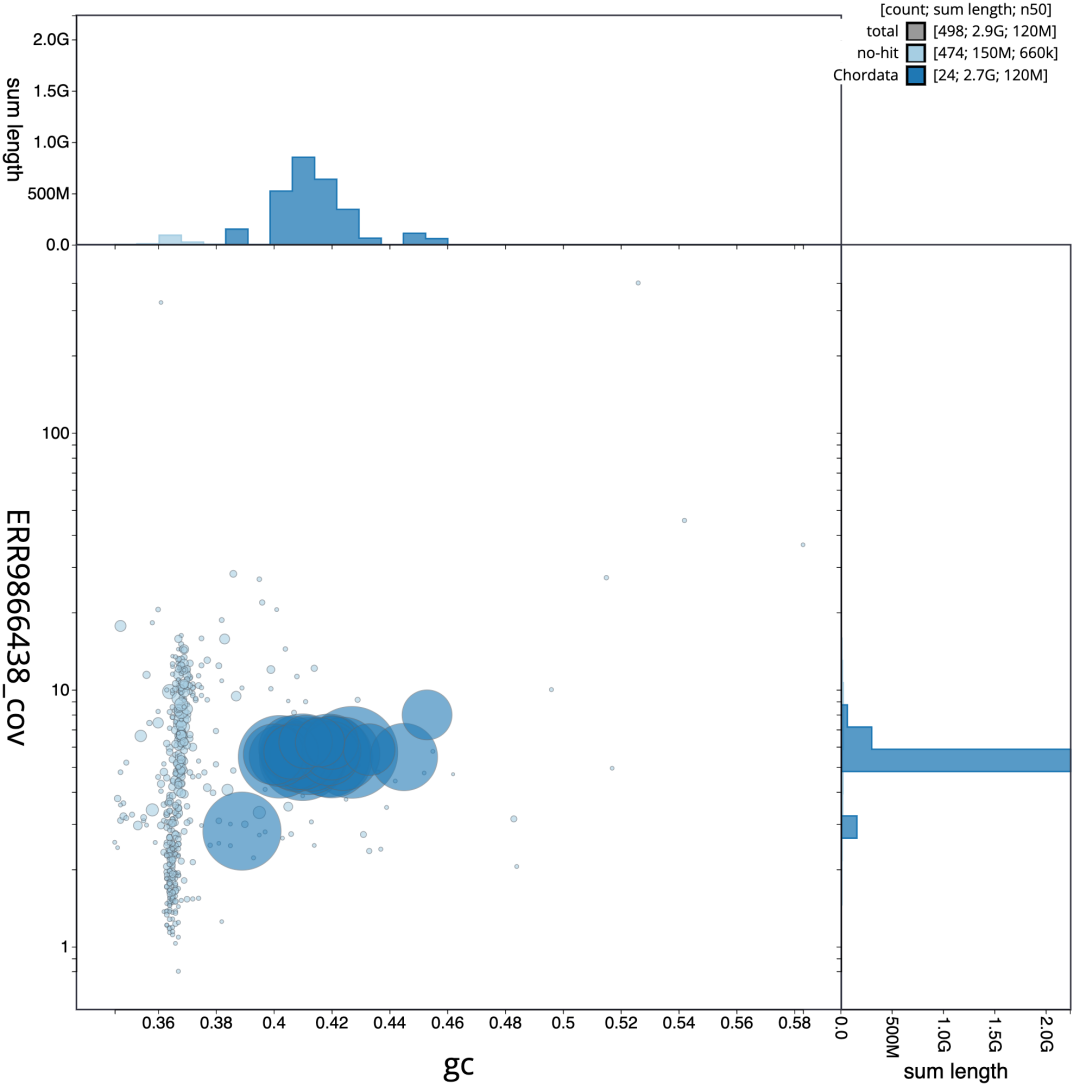
Genome assembly of *Apodemus sylvaticus*, mApoSyl1.1: BlobToolKit GC-coverage plot. Scaffolds are coloured by phylum. Circles are sized in proportion to scaffold length. Histograms show the distribution of scaffold length sum along each axis. An interactive version of this figure is available at https://blobtoolkit.genomehubs.org/view/mApoSyl1.1/dataset/CAMXCG01/blob.

**Figure 4.**
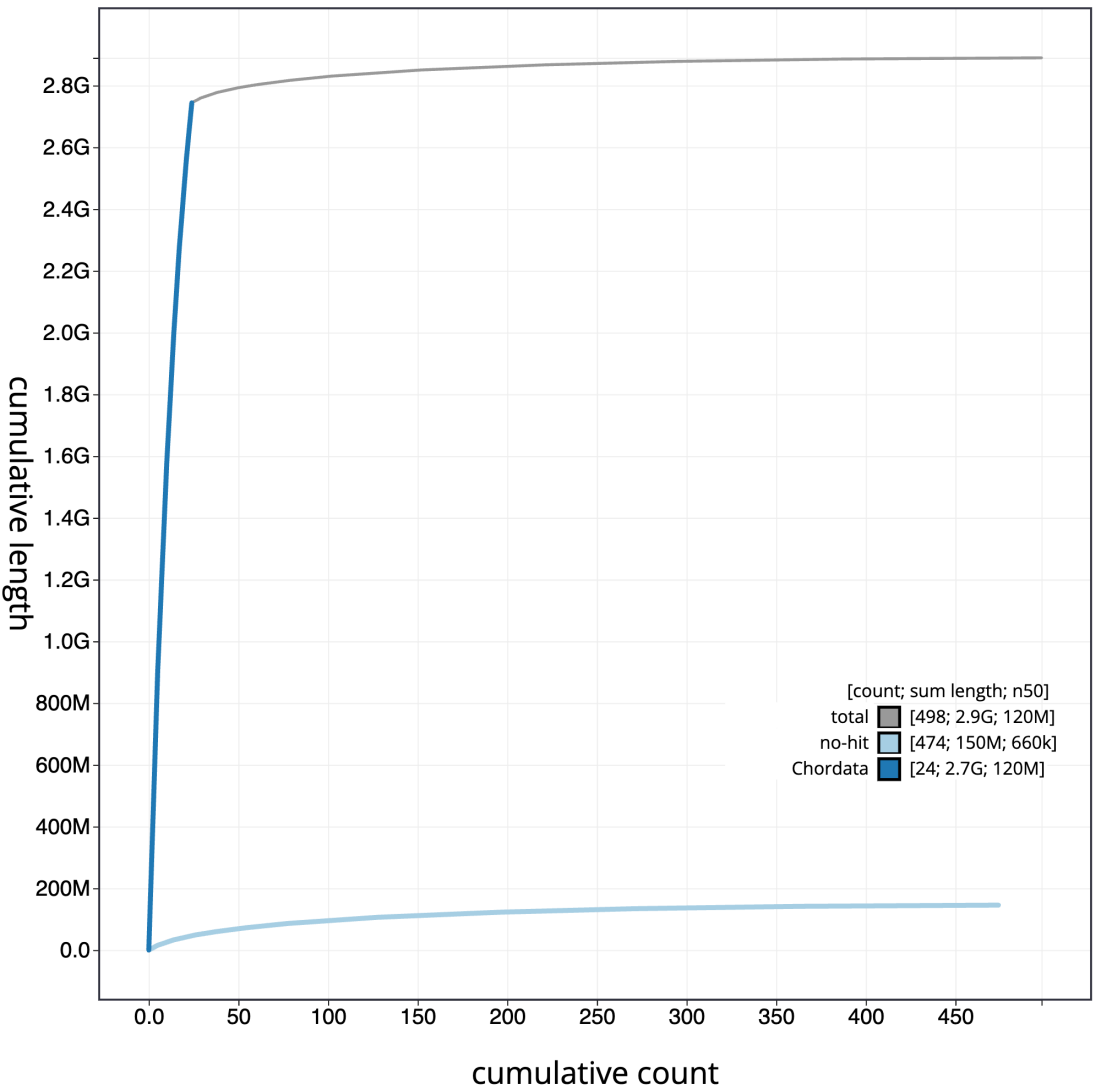
Genome assembly of *Apodemus sylvaticus*, mApoSyl1.1: BlobToolKit cumulative sequence plot. The grey line shows cumulative length for all scaffolds. Coloured lines show cumulative lengths of scaffolds assigned to each phylum using the buscogenes taxrule. An interactive version of this figure is available at https://blobtoolkit.genomehubs.org/view/mApoSyl1.1/dataset/CAMXCG01/cumulative.

**Figure 5.**
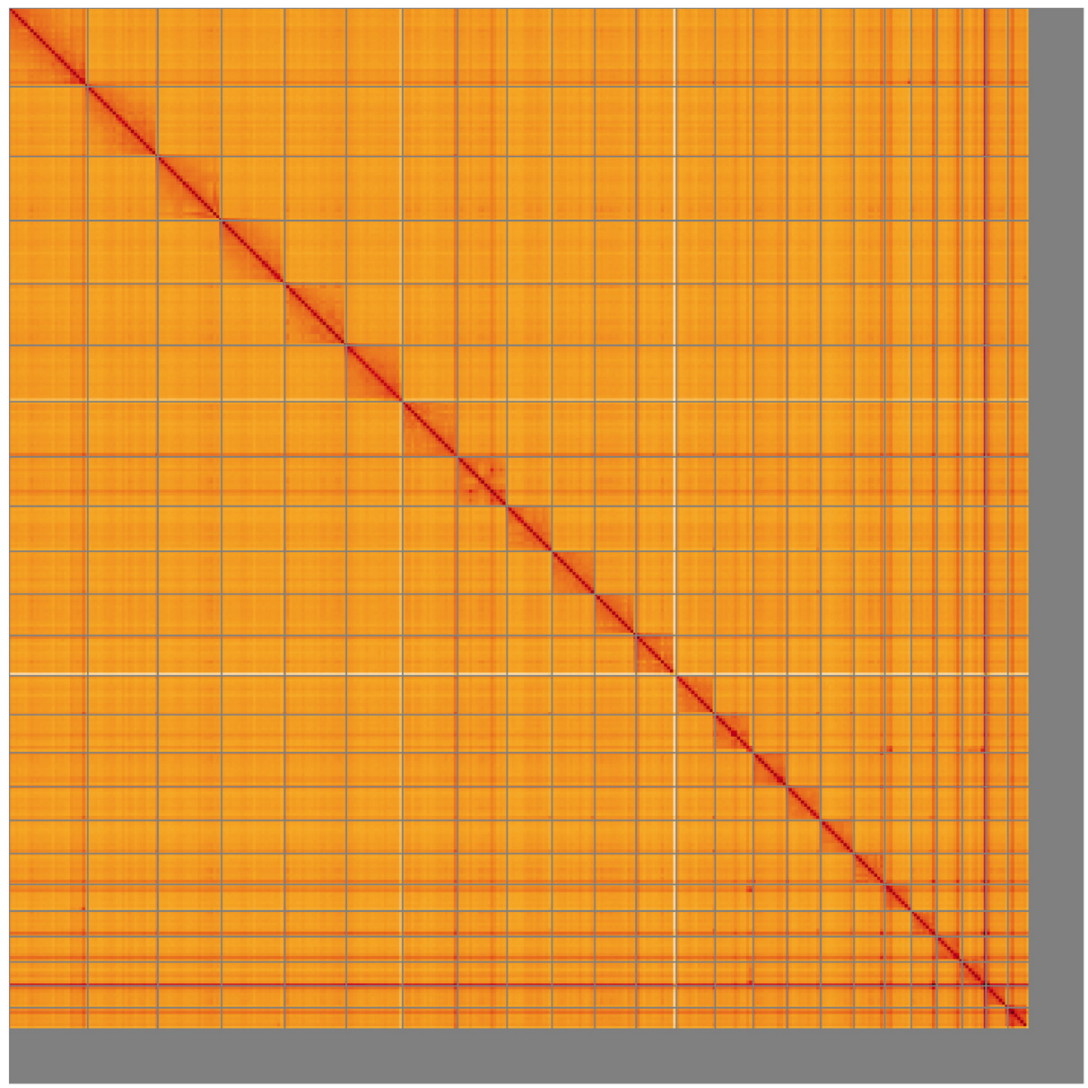
Genome assembly of *Apodemus sylvaticus*, mApoSyl1.1: Hi-C contact map of the mApoSyl1.1 assembly, visualised using HiGlass. Chromosomes are shown in order of size from left to right and top to bottom. An interactive version of this figure may be viewed at https://genome-note-higlass.tol.sanger.ac.uk/l/?d=N_6vvYjRR-SABvzgRaoJkQ.

**Table 2.** Chromosomal pseudomolecules in the genome assembly of *Apodemus sylvaticus*, mApoSyl1.

INSDC accession	Chromosome	Length (Mb)	GC%
OX359297.1	1	211.76	42.5
OX359298.1	2	188.06	41.0
OX359299.1	3	171.66	42.0
OX359300.1	4	170.26	40.0
OX359301.1	5	165.11	42.0
OX359303.1	6	147.57	41.0
OX359304.1	7	132.81	42.5
OX359305.1	8	120.94	41.0
OX359306.1	9	114.97	41.5
OX359307.1	10	111.23	44.5
OX359308.1	11	108.32	40.5
OX359309.1	12	104.47	41.0
OX359310.1	13	102.39	40.5
OX359311.1	14	91.74	42.0
OX359312.1	15	89.43	40.0
OX359313.1	16	89.84	40.0
OX359314.1	17	82.79	42.0
OX359315.1	18	71.5	40.5
OX359316.1	19	68.85	42.0
OX359317.1	20	68.0	41.0
OX359318.1	21	63.78	43.5
OX359319.1	22	59.29	45.5
OX359320.1	23	57.62	41.5
OX359302.1	X	151.77	39.0
OX359321.1	Y	3.45	36.5
OX359322.1	MT	0.02	36.5

The estimated Quality Value (QV) of the final assembly is 55.9 with *k*-mer completeness of 99.99%, and the assembly has a BUSCO v5.3.2 completeness of 96.0% (single = 95.0%, duplicated = 1.0%), using the glires_odb10 reference set ( *n* = 13,798).

Metadata for specimens, spectral estimates, sequencing runs, contaminants and pre-curation assembly statistics can be found at https://tolqc.cog.sanger.ac.uk/darwin/mammals/Apodemus_sylvaticus/.

## Methods

### Sample acquisition and nucleic acid extraction

Two *Apodemus sylvaticus*, a male and a female, were live-caught in baited Sherman traps in Wytham Woods, Oxfordshire (biological vice-county Berkshire), UK (latitude 51.77, longitude –1.34) on 2020-10-28. The animals were collected and identified by Aura Raulo and Sarah Knowles (University of Oxford). A small ear clip was taken from each mouse using a sterilised ear punch, and snap-frozen on dry ice (under Home Office license PPL PB0178858). The specimen used for DNA sequencing was the male *Apodemus sylvaticus* (specimen ID Ox000983, ToLID mApoSyl1), while the female specimen ID Ox000985, ToLID mApoSyl2) was used for Hi-C data. Both animals were released at their site of capture within several hours of trap collection.

DNA was extracted at the Tree of Life laboratory, Wellcome Sanger Institute (WSI). The mApoSyl1 sample was weighed and dissected on dry ice with tissue set aside for Hi-C sequencing. The tissue was disrupted using a Nippi Powermasher fitted with a BioMasher pestle. High molecular weight (HMW) DNA was extracted using the Qiagen MagAttract HMW DNA extraction kit. Low molecular weight DNA was removed from a 20 ng aliquot of extracted DNA using the 0.8X AMpure XP purification kit prior to 10X Chromium sequencing; a minimum of 50 ng DNA was submitted for 10X sequencing. HMW DNA was sheared into an average fragment size of 12–20 kb in a Megaruptor 3 system with speed setting 30. Sheared DNA was purified by solid-phase reversible immobilisation using AMPure PB beads with a 1.8X ratio of beads to sample to remove the shorter fragments and concentrate the DNA sample. The concentration of the sheared and purified DNA was assessed using a Nanodrop spectrophotometer and Qubit Fluorometer and Qubit dsDNA High Sensitivity Assay kit. Fragment size distribution was evaluated by running the sample on the FemtoPulse system.

### Sequencing

Pacific Biosciences HiFi circular consensus and 10X Genomics read cloud DNA sequencing libraries were constructed according to the manufacturers’ instructions. DNA sequencing was performed by the Scientific Operations core at the WSI on Pacific Biosciences SEQUEL II (HiFi) and Illumina NovaSeq 6000 (10X) instruments. Hi-C data were also generated from the mApoSyl2 sample using the Arima2 kit and sequenced on the Illumina NovaSeq 6000 instrument.

### Genome assembly, curation and evaluation

Assembly was carried out with Hifiasm ( Cheng *et al.*, 2021) and haplotypic duplication was identified and removed with purge_dups ( Guan *et al.*, 2020). One round of polishing was performed by aligning 10X Genomics read data to the assembly with Long Ranger ALIGN, calling variants with FreeBayes ( Garrison & Marth, 2012). The assembly was then scaffolded with Hi-C data ( Rao *et al.*, 2014) using YaHS ( Zhou *et al.*, 2023). The assembly was checked for contamination and corrected using the gEVAL system ( Chow *et al.*, 2016) as described previously ( Howe *et al.*, 2021). Manual curation was performed using gEVAL, HiGlass ( Kerpedjiev *et al.*, 2018) and Pretext ( Harry, 2022). The mitochondrial genome was assembled using MitoHiFi ( Uliano-Silva *et al.*, 2023), which runs MitoFinder ( Allio *et al.*, 2020) or MITOS ( Bernt *et al.*, 2013) and uses these annotations to select the final mitochondrial contig and to ensure the general quality of the sequence.

A Hi-C map for the final assembly was produced using bwa-mem2 ( Vasimuddin *et al.*, 2019) in the Cooler file format ( Abdennur & Mirny, 2020). To assess the assembly metrics, the *k*-mer completeness and QV consensus quality values were calculated in Merqury ( Rhie *et al.*, 2020). This work was done using Nextflow ( Di Tommaso *et al.*, 2017) DSL2 pipelines “sanger-tol/readmapping” ( Surana *et al.*, 2023a) and “sanger-tol/genomenote” ( Surana *et al.*, 2023b). The genome was analysed within the BlobToolKit environment ( Challis *et al.*, 2020) and BUSCO scores ( Manni *et al.*, 2021; Simão *et al.*, 2015) were calculated.

Table 3 contains a list of relevant software tool versions and sources.

**Table 3.** Software tools: versions and sources.

Software tool	Version	Source
BlobToolKit	4.1.5	https://github.com/blobtoolkit/blobtoolkit
BUSCO	5.3.2	https://gitlab.com/ezlab/busco
FreeBayes	1.3.1-17-gaa2ace8	https://github.com/freebayes/freebayes
gEVAL	N/A	https://geval.org.uk/
Hifiasm	0.16.1-r375	https://github.com/chhylp123/hifiasm
HiGlass	1.11.6	https://github.com/higlass/higlass
Long Ranger ALIGN	2.2.2	https://support.10xgenomics.com/genome-exome/software/pipelines/latest/advanced/other-pipelines
Merqury	MerquryFK	https://github.com/thegenemyers/MERQURY.FK
MitoHiFi	2	https://github.com/marcelauliano/MitoHiFi
PretextView	0.2	https://github.com/wtsi-hpag/PretextView
purge_dups	1.2.3	https://github.com/dfguan/purge_dups
sanger-tol/genomenote	v1.0	https://github.com/sanger-tol/genomenote
sanger-tol/readmapping	1.1.0	https://github.com/sanger-tol/readmapping/tree/1.1.0
YaHS	yahs-1.1.91eebc2	https://github.com/c-zhou/yahs

### Wellcome Sanger Institute – Legal and Governance

The materials that have contributed to this genome note have been supplied by a Darwin Tree of Life Partner. The submission of materials by a Darwin Tree of Life Partner is subject to the **‘Darwin Tree of Life Project Sampling Code of Practice’**, which can be found in full on the Darwin Tree of Life website here. By agreeing with and signing up to the Sampling Code of Practice, the Darwin Tree of Life Partner agrees they will meet the legal and ethical requirements and standards set out within this document in respect of all samples acquired for, and supplied to, the Darwin Tree of Life Project. 

Further, the Wellcome Sanger Institute employs a process whereby due diligence is carried out proportionate to the nature of the materials themselves, and the circumstances under which they have been/are to be collected and provided for use. The purpose of this is to address and mitigate any potential legal and/or ethical implications of receipt and use of the materials as part of the research project, and to ensure that in doing so we align with best practice wherever possible. The overarching areas of consideration are:

• Ethical review of provenance and sourcing of the material

• Legality of collection, transfer and use (national and international) 

Each transfer of samples is further undertaken according to a Research Collaboration Agreement or Material Transfer Agreement entered into by the Darwin Tree of Life Partner, Genome Research Limited (operating as the Wellcome Sanger Institute), and in some circumstances other Darwin Tree of Life collaborators.

## Data Availability

European Nucleotide Archive: *Apodemus sylvaticus* (wood mouse); Accession number PRJEB53556; https://identifiers.org/ena.embl/PRJEB53556. ( Wellcome Sanger Institute, 2022) The genome sequence is released openly for reuse. The *Apodemus sylvaticus* genome sequencing initiative is part of the Darwin Tree of Life (DToL) project. All raw sequence data and the assembly have been deposited in INSDC databases. The genome will be annotated using available RNA-Seq data and presented through the Ensembl pipeline at the European Bioinformatics Institute. Raw data and assembly accession identifiers are reported in Table 1.

## Author information

Members of the University of Oxford and Wytham Woods Genome Acquisition Lab are listed here: https://doi.org/10.5281/zenodo.4789928.

Members of the Darwin Tree of Life Barcoding collective are listed here: https://doi.org/10.5281/zenodo.4893703.

Members of the Wellcome Sanger Institute Tree of Life programme are listed here: https://doi.org/10.5281/zenodo.4783585.

Members of Wellcome Sanger Institute Scientific Operations: DNA Pipelines collective are listed here: https://doi.org/10.5281/zenodo.4790455.

Members of the Tree of Life Core Informatics collective are listed here: https://doi.org/10.5281/zenodo.5013541.

Members of the Darwin Tree of Life Consortium are listed here: https://doi.org/10.5281/zenodo.4783558.
