# Supplementary material for: The genome sequence of the wood mouse, Apodemus sylvaticus (Linnaeus, 1758)
Source: Wellcome Open Res. 2023 Oct 12;8:442. [Version 1] doi: 10.12688/wellcomeopenres.20001.1 (PMC11294814; doi:10.12688/wellcomeopenres.20001.1)
Supplement: Peer review history

# Reviewer report: Reviewer response for version 1

**DOI:** 10.21956/wellcomeopenres.22147.r89632

**Published:** 2024-08-06

**Authors:** Jakub Skorupski

**Affiliations:** 1 Uniwersytet Szczecinski, Szczecin, West Pomeranian Voivodeship, Poland

The article presents the genome assembly and annotation of *Apodemus sylvaticus*, commonly known as the wood mouse. The study is part of the Darwin Tree of Life Project, aiming to sequence the genomes of all eukaryotic species in the British Isles. The research involved capturing two specimens, extracting high molecular weight DNA, sequencing, and performing various bioinformatics analyses to produce a high-quality genome assembly. The assembly provides significant insights into the genetic composition and structure of the species, offering valuable data for comparative genomics and evolutionary studies.

 The article clearly outlines the rationale behind creating the dataset, emphasizing the importance of sequencing *Apodemus sylvaticus* as part of the broader goal to understand the biodiversity of the British Isles and contribute to the Darwin Tree of Life Project. The authors also highlight the species' ecological significance and the potential insights the genomic data can provide into evolutionary biology and conservation.

 The protocols employed in the study are standard and appropriate for genomic sequencing and assembly. The authors utilized high-quality DNA extraction methods, various sequencing technologies, and advanced bioinformatics tools for assembly and annotation. The detailed description of the procedures, including the use of tools like BlobToolKit and BUSCO, ensures that the work is technically robust.

 The article provides comprehensive details of the methods and materials used, including specifics about DNA extraction, sequencing, and data analysis. The inclusion of software versions and sources further aids in replicability. The study's methodology is meticulously documented, making it possible for other researchers to replicate the work under similar conditions.

 The datasets are presented clearly and are made accessible through various online platforms, including BlobToolKit and the Darwin Tree of Life Project's repositories. Interactive visualizations and comprehensive metadata ensure that the data is usable for further research and analyses.

 While the rationale is clear, a more in-depth discussion on the specific ecological and evolutionary questions the dataset can address would enhance the relevance and context.

 The article successfully meets the standards of scientific reporting, with clear rationale, appropriate protocols, comprehensive methodological details, and accessible data presentation. The study is scientifically sound and provides a valuable resource for the scientific community. The minor improvements suggested are intended to further strengthen the clarity and utility of the work.

Are sufficient details of methods and materials provided to allow replication by others?

Yes

Is the rationale for creating the dataset(s) clearly described?

Yes

Are the datasets clearly presented in a useable and accessible format?

Yes

Are the protocols appropriate and is the work technically sound?

Yes

Reviewer Expertise:

conservation genetics, genomics, conservation biology, ecology

I confirm that I have read this submission and believe that I have an appropriate level of expertise to confirm that it is of an acceptable scientific standard.

# Reviewer report: Reviewer response for version 1

**DOI:** 10.21956/wellcomeopenres.22147.r88284

**Published:** 2024-08-01

**Authors:** Roberto Biello

**Affiliations:** 1 University of Ferrara, Ferrara, Italy; 2 John Innes Centre, Norwich Research Park, Norwich, UK

To the best of my knowledge, the authors report the first chromosome-level genome assembly of the wood mouse, *Apodemus sylvaticus*. This genomic resource will be highly beneficial for research on this species, given its status as a key model organism in evolutionary biology due to its abundance, ease of capture, and ability to be maintained in captivity.

 The authors provide a comprehensive introduction to this species and justify the need for generating a new genome assembly. The sequencing was performed using PacBio HiFi, 10x Genomics, and Hi-C data. The resulting assembly meets and exceeds general benchmarks for high-quality assemblies, including scores for BUSCO, k-mer completeness, and the percentage of the assembly mapped to chromosomes. All raw and analysed datasets are deposited in publicly available repositories.

 I see no need for additional improvements or revisions to the manuscript.

Are sufficient details of methods and materials provided to allow replication by others?

Yes

Is the rationale for creating the dataset(s) clearly described?

Yes

Are the datasets clearly presented in a useable and accessible format?

Yes

Are the protocols appropriate and is the work technically sound?

Yes

Reviewer Expertise:

Population genomics, Conservation, Comparative genomics

I confirm that I have read this submission and believe that I have an appropriate level of expertise to confirm that it is of an acceptable scientific standard.

# Reviewer report: Reviewer response for version 1

**DOI:** 10.21956/wellcomeopenres.22147.r89636

**Published:** 2024-08-01

**Authors:** Xiuguang Mao

**Affiliations:** 1 East China Normal University, Shanghai, China

This manuscript presents a high-quality chromosome-scale assembly for one male *Apodemus sylvaticus*, which will be very useful for studies in ecology and evolutionary biology. I have two minor comment:

- “Most of the assembly sequence was assigned to 25 chromosomal-level scaffolds, representing 23 autosomes and the X and Y sex chromosomes.” In Methods the authors should provide details about how they determine the sex chromosomes, such as by performing genome alignments with previous high-quality genomes or comparing the difference of sequencing coverage between autosomes and sex chromosomes?
- In Table 2 chromosome 16 is longer than chromosome 15 in length. Thus, to make sure that “chromosomes are shown in order of size”, Chr15 and Chr16 should be reordered.

Are sufficient details of methods and materials provided to allow replication by others?

Yes

Is the rationale for creating the dataset(s) clearly described?

Yes

Are the datasets clearly presented in a useable and accessible format?

Yes

Are the protocols appropriate and is the work technically sound?

Yes

Reviewer Expertise:

Comparative genomics, genome assembly, evolutionary biology, speciation, bats

I confirm that I have read this submission and believe that I have an appropriate level of expertise to confirm that it is of an acceptable scientific standard.

# Reviewer report: Reviewer response for version 1

**DOI:** 10.21956/wellcomeopenres.22147.r89633

**Published:** 2024-07-31

**Authors:** Daniel Garcia-Souto

**Affiliations:** 1 University of Santiago de Compostela, Santiago de Compostela, Galicia, Spain

The presented genome assembly of the wood mouse, *Apodemus sylvaticus*, showcases an exceptional quality both in continuity and annotation. The assembly spans into 25 scaffolded chromosomal pseudomolecules, demonstrating a high level of continuity. Furthermore, the inclusion of both X and Y sex chromosomes and the mitochondrial genome, reflects the most complete version of the genome of this species.

 This genome assembly is of particular significance due to the known presence of accessory chromosomes and Robertsonian translocations within populations of this organism, which make *Apodemus sylvaticus* an interesting subject for genetic and evolutionary studies. The high-quality assembly provided here will facilitate further research into these processes, enhancing our understanding of genome architecture and evolution in rodent species.

Are sufficient details of methods and materials provided to allow replication by others?

Yes

Is the rationale for creating the dataset(s) clearly described?

Yes

Are the datasets clearly presented in a useable and accessible format?

Yes

Are the protocols appropriate and is the work technically sound?

Yes

Reviewer Expertise:

Cytogenetics, Genomics, Evolution, Karyotyping

I confirm that I have read this submission and believe that I have an appropriate level of expertise to confirm that it is of an acceptable scientific standard.
